# The contribution of linear perspective cues and texture gradients in the perceptual rescaling of stimuli inside a Ponzo illusion corridor

**DOI:** 10.1371/journal.pone.0223583

**Published:** 2019-10-10

**Authors:** Gizem Y. Yildiz, Irene Sperandio, Christine Kettle, Philippe A. Chouinard

**Affiliations:** 1 Department of Psychology and Counselling, School of Psychology and Public Health, La Trobe University, Melbourne, Australia; 2 School of Psychology, University of East Anglia, Norwich, United Kingdom; 3 Department of Pharmacy and Biomedical Sciences, School of Molecular Sciences, La Trobe University, Melbourne, Australia; SWPS University of Social Sciences and Humanities, POLAND

## Abstract

We examined the influence of linear perspective cues and texture gradients in the perceptual rescaling of stimuli over a highly-salient Ponzo illusion of a corridor. We performed two experiments using the Method of Constant Stimuli where participants judged the size of one of two rings. In experiment 1, one ring was presented in the upper visual-field at the end of the corridor and the other in the lower visual-field at the front of the corridor. The perceived size of the top and bottom rings changed as a function of the availability of linear perspective and textures. In experiment 2, only one ring was presented either at the top or the bottom of the image. The perceived size of the top but not the bottom ring changed as a function of the availability of linear perspective and textures. In both experiments, the effects of the cues were additive. Perceptual rescaling was also stronger for the top compared to the bottom ring. Additional eye-tracking revealed that participants tended to gaze more in the upper than the lower visual-field. These findings indicate that top-down mechanisms provide an important contribution to the Ponzo illusion. Nonetheless, additional maximum likelihood estimation analyses revealed that linear perspective fulfilled a greater contribution in experiment 2, which is suggestive of a bottom-up mechanism. We conclude that both top-down and bottom-up mechanisms play important roles. However, the former seems to fulfil a more prominent role when both stimuli are presented in the image.

## Introduction

If two objects at different distances subtend the same visual angle on the retina then the object located at the furthest distance is physically larger as a proportion of distance from the other in a manner obeying Euclidian geometry [1]. Size constancy mechanisms operate so that our visual system considers these physical realities and enables us to perceive objects as having the same size regardless of changes in viewing conditions. To maintain size constancy, the visual system estimates the distance between the object and the eyes from many sources of depth cues and perceptually rescales the retinal input about the size of the object [2, 3]. Linear perspective is one of many pictorial depth cues that the visual system uses to estimate depth [4]. The visual system estimates greater depth when two lines on the retina converge closer together. Another important pictorial depth cue is texture gradient. The retinal size of uniform texture elements, such as stones, shrink with distance. Consequently, the visual system estimates greater depth where texture gradients are smaller. Artists apply this knowledge to create illusory depth on a 2D image to trick us in perceiving depth and size differences [5].

**Fig 1:**
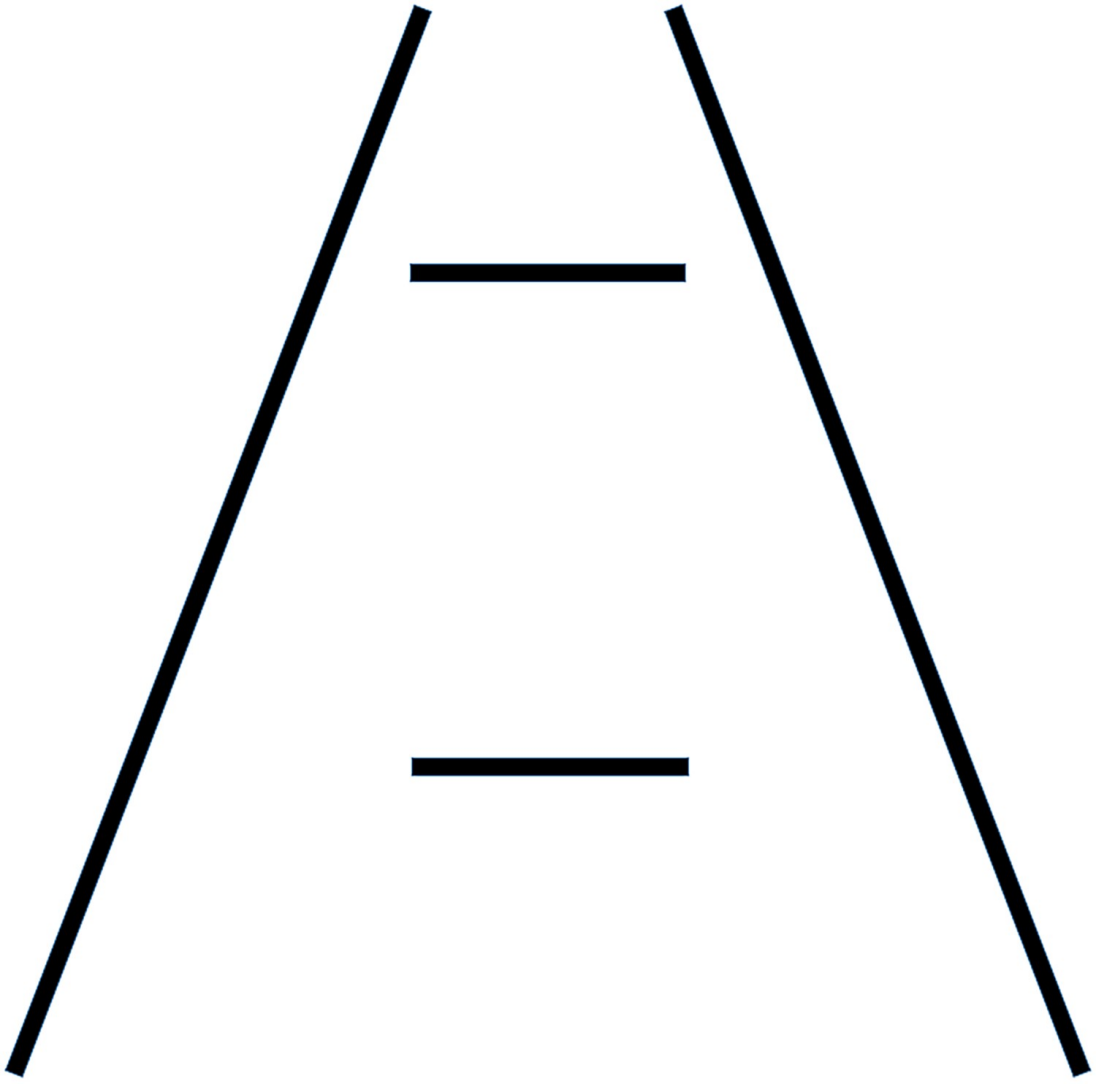
Ponzo illusion. The figure provides an illustration of the Ponzo illusion in its classical configuration with two simple lines converging upwards and two horizontal lines in the centre that are identical in length but appear different.

In the Ponzo illusion, two physically identical stimuli appear to be different from each other when placed over the top and bottom sections of converging contextual lines that emulate a vanishing point, like the converging lines of a railway track or the converging walls of a corridor [6]. Specifically, the top stimulus where the contextual lines converge appears to be larger than the bottom one (Fig 1). Misapplied constancy scaling theory is one of several theories that explains the Ponzo illusion. According to the theory, the pictorial depth information in the background will rescale the size of objects in such a manner that those that appear further are perceived larger [2, 7, 8]. With regards to the Ponzo illusion, the visual system interprets the converging lines as parallel lines receding into the distance, as linear perspective cues do in the real world, and perceptually rescales stimuli as a function of how far they appear to be [9, 10]. Many have argued that this perceptual rescaling is driven by top-down modulation arising from knowledge acquired from everyday life experiences about how linear perspective cues inform the brain about how far objects are (e.g. watching trains on railway tracks, cars on highways, etc.) [2, 8].

However, to this day, the relative contribution of the linear perspective cues and texture gradients to the magnitude of illusory size perception in the context of the Ponzo illusion remains debated despite preliminary research [11, 12]. Leibowitz et al. [11] reported that applying only texture gradients was twice as strong as when only applying linear perspective cues. Moreover, they showed an additive effect when the texture gradients were combined with the linear perspective cues. Specifically, texture gradients alone and linear perceptive cues alone perceptually rescaled the size of stimuli by 20% and 10%, respectively. The presence of both cues perceptually rescaled the size of stimuli by 30%. These additive effects suggested to Leibowitz et al. [11] that the magnitude of the Ponzo illusion is closely dependent upon the availability of the pictorial depth cues. We reason that these effects were driven largely by top-down mechanisms. If the two types of depth cues influenced size perception by separate channels in a bottom-up manner without integration then one might expect to find the same degree of perceptual rescaling with both cues present as when only the stronger of the two cues is present rather than the additive effects seen in the Leibowitz et al. [11] study. This same line of reasoning is also explained elsewhere [13].

However, the study by Leibowitz et al. [11] is not without controversy. Fineman and Carlson [12] questioned whether their choice of a background image for the texture gradient condition included only texture gradients as a depth cue. It could have been the case that this condition also offered linear perspective in how the textures were arranged. To help resolve this issue, the authors tested the contribution of texture gradients using Gibson’s [14] dot patterns. Contrary to the results obtained by Leibowitz et al [11], they demonstrated that texture gradients had little to no effect on perceptual rescaling. Based on these results, the authors favoured a bottom-up explanation of the Ponzo illusion.

In line with Fineman and Carlson’s findings, others have reported that the manipulation of texture gradients does not affect perceptual judgments of size [15, 16]. Similarly, studies examining the effects of texture gradients on perceived depth have shown that the visual system is less sensitive to the manipulation of texture gradients compared to the manipulation of linear perspective cues [17–20]. For instance, Zhang [20] found that perceptual judgments of depth did not change as a function of the availability of texture gradients in an immersive driving simulator, suggesting once again that this kind of pictorial depth cue is not relevant for the perceptual rescaling of size.

So far, the literature reviewed has yielded mixed results. The mixed results could have arisen from a fundamental limitation present in all of the aforementioned studies. Namely, the background images that incorporated the two cues were not graphically additive to those consisting of the presentation of only one. In other words, there was no real graphical subtraction or addition of cues in the background images across conditions. Instead, the images that were previously used consisted of completely different images with little similarities amongst each other. The only study that we are aware of that has used a more systematic approach is one performed by Rennig, Karnath, and Huberle [13]. The authors examined the effects of linear perspective cues and texture gradients on the perceived size of Kanizsa triangles, an illusory contour, over a Ponzo-like background of a corridor. The two pictorial depth cues affected the perception of the Kanizsa triangles differently. Namely, the Kanizsa triangle that appeared further away (i.e. the top one) was perceived larger in the linear perspective but not in the texture gradient condition.

**Fig 2:**
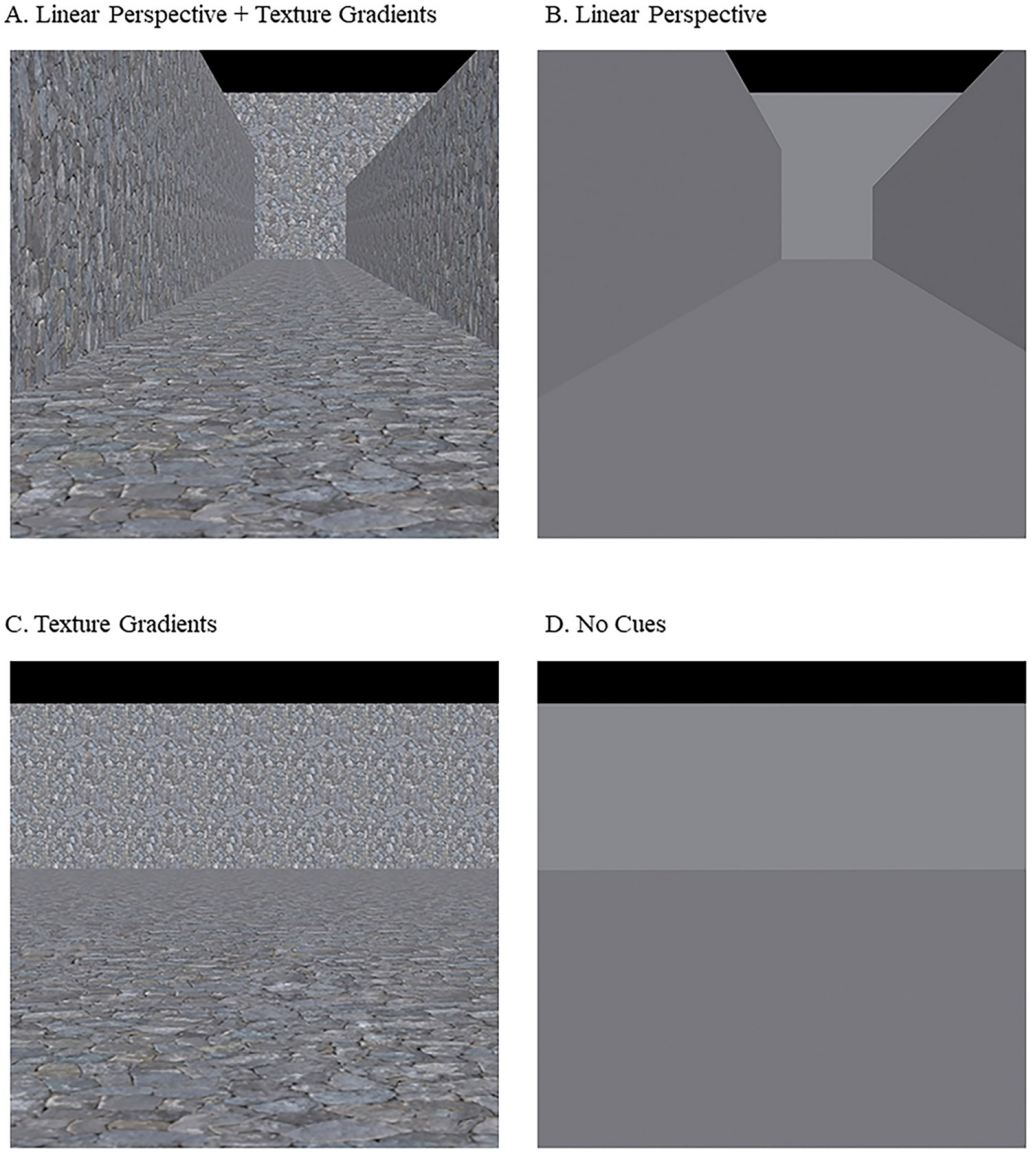
Background images in the present study. A. Ponzo-like illusion display of a hallway with stones (textures) and walls (linear perspective cues). B. Ponzo-like illusion display of walls (linear perspective cues). C. Ponzo-like illusion display of a hallway with stones (texture gradients). D. Control background without depth cues.

In the present study, we graphically added and removed linear perspective cues and texture gradients in a Ponzo-like illusory display of a hallway (Fig 2) to determine how these manipulations might affect the perceived size of stimuli. The manipulation of these pictorial depth cues allowed us to examine the relative contribution of top-down and bottom-up mechanisms indirectly. We propose that if the two cues perceptually rescale the stimuli separately and produce an additive effect when they are combined together, then we can infer that top-down mechanisms fulfil an important contribution to the illusion. This is because both cues afford predictive values about depth and their presence should influence size perception in an integrative manner on the basis of these affordances. On the other hand, we reason that if only a subset of cues exerts an effect, despite their predictive value, or that both cues do not exert an additive effect, then we can infer that integration was minimal and that bottom-up mechanisms by separate channels play an important role in driving the illusion. It is likely that both mechanisms fulfil a role given the evidence so far. The study tries to shed additional light on their respective contributions.

To examine the relative importance of each, we conducted two experiments where participants judged the size of a standard stimulus over one of four different backgrounds ((A) linear perspective cues + textures, (B) linear perspective cues, (C) textures or (D) no cues) (Fig 2). In experiment 1, we presented both the standard and comparison stimuli over the same background image. We hypothesised an illusion that varied in strength as a function of the availability of linear perspective cues and texture gradients. The presentation of the standard and comparison stimuli over the same background is common. However, under this configuration, size-contrast effects can further increase the perceived differences in size that are driven by the pictorial cues. To help minimise size-contrast effects so that we could better isolate the effect of pictorial cues, we presented the comparison stimulus outside of the background so that only one ring was presented over the background in experiment 2. In this case, we hypothesised that the size illusion would be weaker but still present.

In addition, we recorded eye positioning during the task. To the best of our knowledge, eye movements under free gaze conditions have never been measured before in previous investigations of the Ponzo illusion. We hypothesised that participants would spend different amounts of time looking at the upper and lower sections of the Ponzo illusion background. Specifically, we hypothesised that durations in fixation would be larger for the upper visual field since depth cues often draw our attention towards this field in the real world [21]. Moreover, it is well reported that attending to particular parts of an illusion display increases its strength [21–24]. For these reasons, we hypothesised that illusion strength would increase in conditions where participants direct their attention more to the upper visual field.

## Method

### Participants

Sixteen participants (*M*_*Age*_ = 20.43 years, *SD* = 2.31, 8 males) participated in experiment 1 and sixteen participants (*M*_*Age*_ = 23.44 years, *SD* = 9.52, 6 males) participated in experiment 2. All had either normal or corrected-to-normal vision. Prior to the experiments, each participant’s visual acuity, stereo-acuity, and colour vision were measured using the Snellen Chart [25], Randot Contour Circles Test [26], and Ishihara’s Test for Colour Deficiency [27]. Visual acuity was 20/25 or better in each eye and stereo acuity was 70 arcsec (0.02 arcdeg) or less for all participants. None of the participants were colour blind. Written informed consent was obtained from each participant before the experiment. At the end of the experiment, participants received gift cards to compensate for their time and any inconveniencies. The study was carried out in accordance with the Declaration of Helsinki and approved by the La Trobe Human Ethics Committee.

### Procedures

Stimuli were presented on an ASUS VG248QE (Taipei, Taiwan) 24" monitor driven by MATLAB (MathWorks, Natick, MA, USA) and Psychtoolbox, Version 3 [28, 29]. The monitor was placed 76 centimetres away from the chin and forehead rest. It was set to a 120 Hz refresh rate with 1920 x 1080 display resolution on a Dell T1700 running Windows 10. Button responses were recorded with a model RB-840 Cedrus Response Pad (Cedrus Corporation, San Pedro, California, USA). For a subset of participants (8 per experiment), we recorded eye positioning using a portable Tobii TX 300 eye-tracker at a sampling rate of 300 Hz (Tobii AB, Stockholm, Sweden). The eye-tracker was placed 60 centimetres away from the chin and forehead rest. Eye-tracking was not performed in everybody due to limited access to this system.

The size perception of two 2-dimensional (2D) red (R = 200, G = 0, B = 0) rings with a thickness of 0.16 degrees was evaluated using the Method of Constant Stimuli [30]. One of the rings was designated as the standard and the other as the comparison stimulus. The standard ring, and the comparison ring in experiment 1, was presented over one of the following backgrounds: (1) linear perspective cues + textures, (2) linear perspective cues, (3) textures, or (4) no cues (Fig 2).

**Fig 3:**
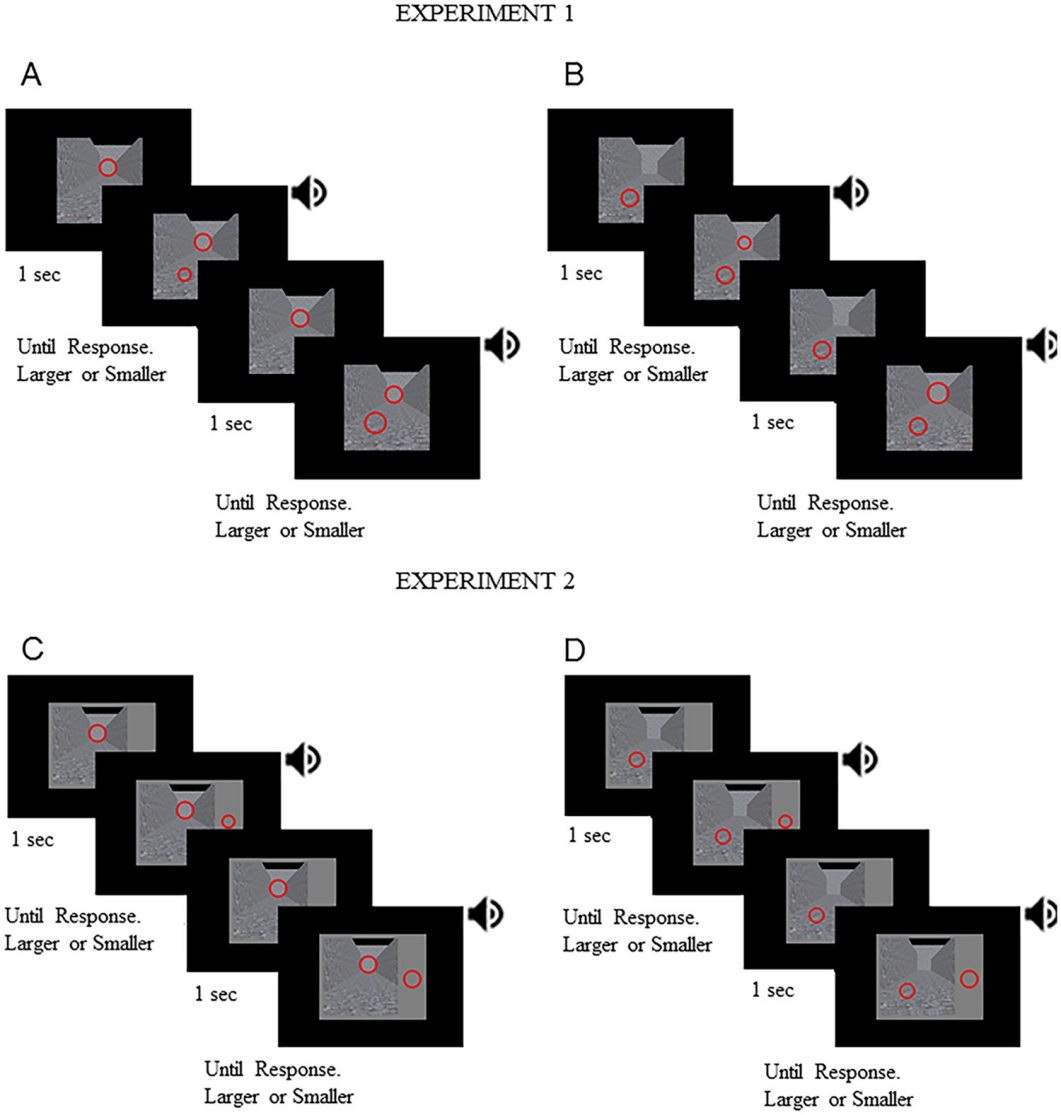
Stimuli and procedures. Illustration of stimuli and procedures that were used in the linear perspective + texture gradient background in experiments 1 and 2 (A-B and C-D, respectively). For the top standard ring block, the top standard ring was shown for 1 sec followed by an alerting sound cue that signalled the presentation of the bottom comparison ring (A and C for experiments 1 and 2, respectively). For the bottom standard ring block, the bottom standard ring was shown for 1 sec followed by an auditory alerting cue that signalled the presentation of the top comparison ring (B and D for experiments 1 and 2, respectively). In experiment 1, both the standard and comparison stimuli were presented over the same background image (A and B). In experiment 2, the comparison ring was presented outside of the background image within the grey area (C and D). The speaker symbols represent the presentation of the auditory alerting cue.

In experiment 1, both the standard and comparison stimuli were presented over the same background image (12.6 × 12.6 degrees) (Fig 3A and 3B). The standard and comparison rings were separated by a vertical distance of 5.24 degrees and a horizontal distance of 3.26 degrees. In experiment 2, we used similar stimuli and followed similar procedures as we used in experiment 1 except for three differences. First, in experiment 2, the background image subtended the same visual angle but was presented over a larger grey area (14 × 18 degrees). Second, the comparison ring was presented outside of the background image within the grey area (Fig 3C and 3D). Third, in experiment 2, the standard and comparison rings were separated by a horizontal distance of 10.74 degrees for the bottom standard ring and 7.52 degrees for the top standard ring. The vertical distance between the standard and comparison rings was 2.62 degrees. In both experiment 1 and experiment 2, the rings were always presented in the same configuration.

For each experiment, there were eight blocks. Each block corresponded to a different condition. Specifically, there was a block for each background with the standard stimulus on the top portion of the image and there was a block for each background with the standard stimulus on the bottom portion of the image (Fig 3). Half of the participants performed the blocks with the standard stimulus on top before performing the blocks with the standard stimulus at the bottom, while the other half of the participants did the reverse. The order of background presentations was randomised for each participant. The standard ring was always kept constant at 2.1 degrees in diameter on all trials and the comparison ring ranged in diameter from 1.64 degrees to 2.54 degrees in 10 increments with 0.1 degrees difference. Each comparison size was shown 10 times in a single background per block. Thus, there were 100 trials per block. The order of trials within each block was randomised.

The graphical reduction of pictorial depth cues was accomplished by removing linear perspective and / or texture cues from a 3D scene of a hallway and walls created in Autodesk 3ds Max (Autodesk, Inc., San Rafael, CA, USA), a program that is currently frequently used to create virtual environments. The following specifications were used to create the background images. The left and right side walls had a length of 1,800 cm and were used as linear perspective cues onto a hallway with a back wall and floor. The walls on the side had different heights to reduce the possibility of the image popping out instead of going into the distance. The left side wall had a height of 195 cm while the right side wall had a height of 130 cm. The back wall with a width of 2,540 cm and a height of 290 cm was placed at the end of a floor that have a length of 1,800 cm and a width of 2,540 cm. The bottom standard stimulus was presented 130 cm away from the virtual camera while the top standard stimulus was presented 1,300 cm away from the virtual camera.

A high-resolution seamless rock wall image was used as the texture cue. Depth information was increased with bump and specular maps of the rock wall. The bump and specular maps of the seamless rock wall image were created in Adobe Photoshop (Adobe Systems Incorporated, San Jose, CA, USA) and assigned to each textured wall. The textured 3D scene of a hallway and walls was used as the background image with linear perspective cues and texture gradients (Fig 2A). Three more background images were rendered by removing linear perspective cues and / or textures. The background image that covered only texture cues was obtained by removing the side walls (Fig 2C) and the image that covered only linear perspective cues was obtained by removing all texture gradients in Autodesk 3ds Max (Fig 2B). Finally, a control background image without depth cues was created by removing both the linear perspective cues and texture gradients, which served as a baseline background (Fig 2D). To assign a colour to the non-textured backgrounds, we measured the average colour of each textured wall in Adobe Photoshop and assigned their average colour in Autodesk 3ds Max. The background images were derived from taking pictures of the virtual environment using a virtual camera placed 1,800 cm from the back wall. The settings of the virtual camera consisted of a full frame of 35 mm, a focal length of 29 mm, and an aperture of f/8. Global lighting of the virtual environment simulated daylight (6,500 K). The centre of the virtual camera from the floor of the hallway was approximately the same height (35 cm) as the participant’s eyes from the testing table (32 cm). All the rendered background images were cropped in Adobe Photoshop.

The participants were provided with 4 practice trials at the start of each block. The eye-tracker was calibrated with a 9-point calibration display at the beginning of each block for the participants who had eye tracking. For both the practice and experimental trials, the participants were asked to judge whether the comparison ring was smaller or larger than the standard ring. Fig 3 illustrates the order of events in a given trial. The standard ring was always presented on the background. Each trial began with a 60 ms auditory alerting cue whenever the comparison ring was presented. The comparison ring was displayed until participants judged whether it was larger or smaller than the standard ring by pressing a button. After button pressing, the comparison ring disappeared before the next trial began one second later. For every trial, the eye tracker collected data from trial onset until a response was made. A break was provided at the end of each block.

### Statistical analyses

We created psychometric curves for each condition in each participant based on their responses. This was done by counting the number of times the participant reported the comparison stimulus as appearing “larger” than the standard one at each increment. Using the following logistic function, we calculated the probability (*P*) of the participant reporting the comparison stimulus at each increment (0.1 degrees) as appearing larger than the standard stimulus:

$$P(x)=\frac{e^{b0+b1x}}{1+e^{b0+b1x}}$$

Where *b0* and *b1* are coefficient estimates based on an initial general linear model (binary logit) fit. From this function, the PSE was calculated as *P* = 0.5, representing how large the comparison stimulus needed to be for the participant to judge this stimulus as having the same apparent size as the standard stimulus. The resulting curves fit well for the different conditions in each individual in experiments 1 (*r*(6) ranged between .734 − .989) and 2 (*r*(6) ranged between .762 − .979). These resulting PSE values were used for the following statistical analyses in each experiment (see S1 Fig in Supplementary Materials to see the mean PSE curves).

To verify whether or not the standard stimulus in a given condition was perceived differently than its physical size, a one-sample *t*-test against the physical size of the standard ring (100 pixels) was performed. The Bonferroni method was used to correct for multiple comparisons. There were eight one-sample *t*-tests per experiment. Thus, to report the Bonferroni-corrected *p* values (*p*_corr_), we multiplied the observed *p* value (*p*_uncorr_) by the number of comparisons made (i.e., *p*_corr_ = *p*_uncorr_ × 8).

To test the effects of linear perspective cues and texture gradients on the perceived size of the top and bottom rings, a 2 × 4 repeated-measures analysis of variance (ANOVA) with Visual Field ((1) Top Ring, (2) Bottom Ring) and Background ((1) linear perspective cues + textures, (2) linear perspective cues, (3) textures or (4) no cues) as factors was conducted. Greenhouse-Geisser corrections were applied when the assumption of sphericity was not met according to a Mauchly’s sphericity test.

We further analysed the contributions of linear perspective cues and texture gradients for the top and bottom rings based on a maximum likelihood estimation (MLE) model. According to this model, the visual system optimally combines visual cues by taking the reliability of each cue into account [30–32]. The model is based on two assumptions: (1) lower variance in the data is seen when a visual cue is highly reliable and (2) the visual system gives more importance to highly reliable cues when combining information from different cues. To estimate the relative contributions of linear perspective cues and texture gradients in the perceptual rescaling of size, we computed the weighted linear summation of PSE measurements for texture (S_texture_) and linear perspective (S_linear perspective_) backgrounds from their standard deviations using the following formulas:

$$w_{\text{linear}}=(1/\left(\sigma_{\text{linear}\;\text{perspective}}{)}^{2}\right)/(1/\left(\sigma_{\text{linear}\;\text{perspective}}{)}^{2}+1/{\left(\sigma_{\text{textures}}\right)}^{2}\right)$$

$$w_{\text{texture}}=\left(1/{\left(\sigma_{\text{texture}}\right)}^{2}\right)/\left(1/{(\sigma }_{\text{linear}\;\text{perspective}}{)}^{2}+1/{\left(\sigma_{\text{textures}}\right)}^{2}\right)$$

$${\text{S}}_{\text{lineart}+\text{texture}}=w_{\text{linear}\;\text{perspective}\;}{\text{S}}_{\text{linear}\;\text{perspective}}+w_{\text{texture}\;}{\text{S}}_{\text{texture}}$$

Solving for *w* in each experiment provided the respective contributions of linear perspective cues and texture gradients.

To analyse the eye-tracking data, areas of interest (AOIs) were defined as a 5 × 5 cm (3.78 × 3.78 degrees) square region centred on the standard and comparison rings. AOIs were defined using Tobii Studio eye tracking software prior to data collection (Tobii Technology, Inc). During data collection, the eye-tracking system tabulated whether or not eye gaze was directed in each AOI at every frame lasting 3.3 milliseconds. The number of frames with fixation was then computed for each AOI off-line and converted to seconds to calculate fixation durations. These fixation durations represent the sum (not the average) of all trials. Each condition had the same number of trials and the results indicated that there were no differences in trial durations (which ended when participants made a response) between conditions (see below)–enabling us to compare this measure across conditions. A 2 × 2 × 4 repeated measures ANOVA with Area of Interest ((1) Standard, (2) Comparison), Visual Field ((1) Top Standard Ring Block, (2) Bottom Standard Ring Block) and Background ((1) linear perspective cues + textures, (2) linear perspective cues, (3) textures or (4) no cues) as factors was conducted.

Post-hoc pairwise comparisons using Tukey’s honest significance difference (HSD) method, which corrected for multiple comparisons, were conducted to further examine interactions and effects found significant by all ANOVAs performed on the PSEs and fixation durations. Unless specified otherwise, all reported *p* values were corrected for multiple comparisons and were based on an alpha level of .05.

## Results

### Experiment 1

#### Points of subjective equality (PSEs)

**Fig 4:**
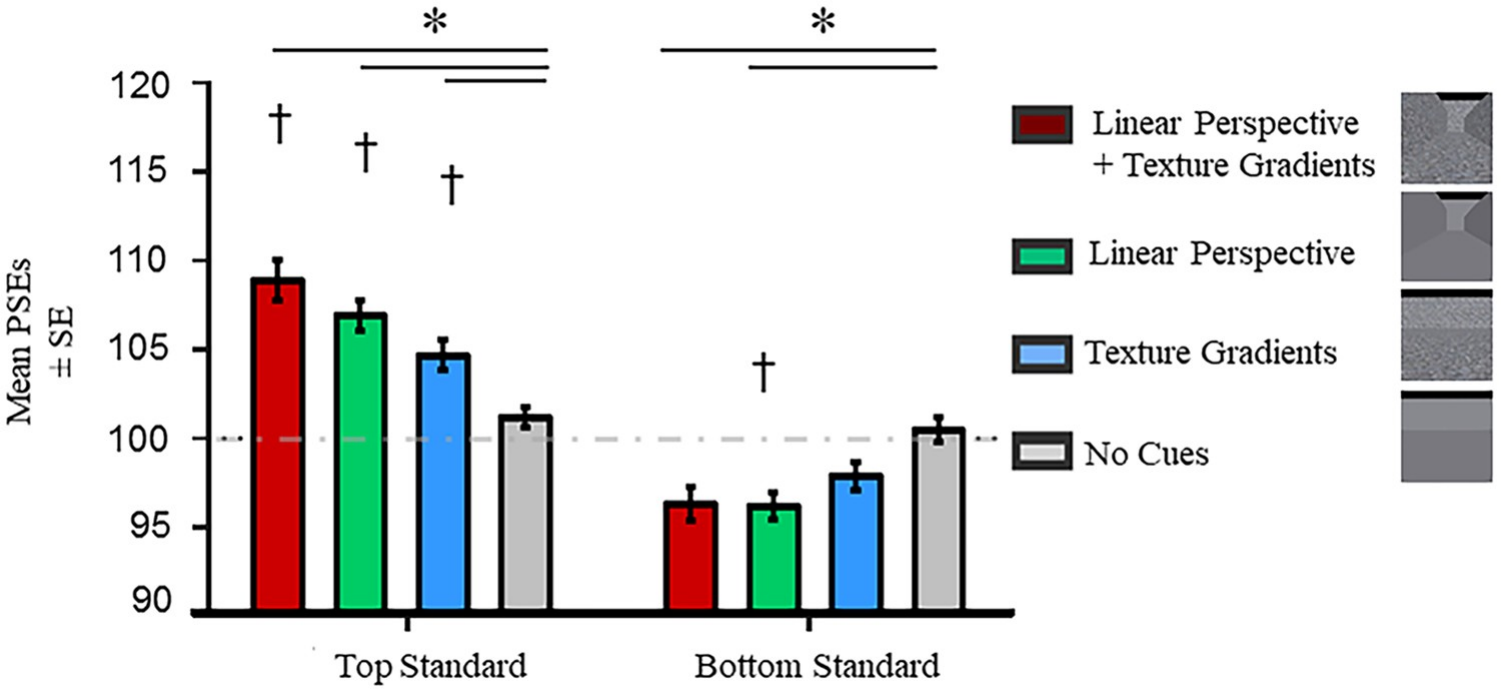
PSEs in experiment 1. Asterisks (*) represent significant differences at *p* < .05 after Tukey’s HSD corrections were made for multiple comparisons. Daggers (†) represent significant differences from the physical size (100 pixels) of the standard ring at *p* < .05 after Bonferroni corrections were made for multiple comparisons. The horizontal dashed line denotes the physical size of the standard ring. PSEs were computed from psychometric functions that best fit the data. Error bars represent the standard errors around the mean for within subjects contrasts. These error bars were calculated using procedures described by O’Brien and Cousineau [33].

Fig 4 shows the mean PSEs for each background for the top and bottom standard ring blocks. Table 1 provides descriptive statistics. To determine if the different background conditions exerted a change in perception relative to retinal information, we compared the PSEs of each background against the physical size of the standard ring (100 pixels) with one sample *t*-tests. One sample *t*-tests revealed that the top standard ring was perceived larger than its physical size across all backgrounds with pictorial depth cues (all *p* ≤ .008) while the bottom standard ring was perceived smaller than its physical size when it was presented with linear perspective cues only (*p* = .016). All significant shifts in PSEs were in the expected direction. Namely, PSEs were greater and lower than 100 pixels when the top and bottom rings was the standard ring, respectively.

**Table 1:** Descriptive statistics for PSEs in experiment 1. A series of independent samples *t*-tests, which were corrected for multiple comparisons using the Bonferroni method (*p*_corr_), on the PSE values for each condition was performed between participants with (With Eye-Tacker) and without (No Eye-Tracker) eye-tracking.

	Group	All Participants (N = 16)		No Eye-Tracker (n = 8)		With Eye-Tracker (n = 8)				
		*M*	*SD*	*M*	*SD*	*M*	*SD*	*t*	*p*_uncorr_	*p*_corr_
**Top Ring**	Linear+Texture	109.2	4.48	106.9	3.42	111.5	4.39	-2.34	0.034	0.272
	Texture	104.9	4.03	103.7	2.64	106	4.98	-1.15	0.269	> .999
	Linear	107.2	4.22	106.2	4.83	108.2	3.54	-0.95	0.356	> .999
	No Cues	101.3	3.09	100.1	2.07	102.5	3.57	-1.70	0.112	0.896
**Bottom Ring**	Linear+Texture	96.4	5.22	95.3	2.17	97.5	7.14	-0.82	0.428	> .999
	Texture	98.0	3.48	98.2	2.6	97.7	4.37	0.28	0.787	> .999
	Linear	96.3	4.01	95.9	1.16	96.6	5.73	-0.35	0.734	> .999
	No Cues	100.6	3.6	99.8	1.67	101.4	4.85	-0.87	0.401	> .999

The PSEs for the top and bottom rings in each of the four backgrounds were compared with each other. An interaction was observed between Visual Field and Background (*F* (3, 45) = 25.68, *p* < .001). Main effects of Visual Field (*F* (1, 15) = 52.08, *p* < .001) and Background (*F* (3, 45) = 3.30, *p* = .029) were also significant. To further examine the interaction, we conducted Tukey’s HSD pairwise comparison tests. These tests showed that the size of the top ring was consistently perceived larger on backgrounds with depth cues than the one without any cues (all *p* ≤ .028) while the size of the bottom ring was perceived smaller on the backgrounds with linear perspective cues compared to the one without any cues (both *p* ≤ .006). There were no differences in the perceived size of the rings when placed on the background with only linear perspective cues versus the one with only texture gradients (both *p* ≥ .394). Taken together, the presence of depth cues affected the perceived size of the top and bottom rings. S1 Table in the supplementary materials provides the results for all pairwise comparisons examined.

We repeated the above ANOVA on the absolute shifts in PSEs (**|**100 –PSE**|**) to confirm if the above interaction in PSEs was driven more strongly by the top ring relative to the bottom ring. The ANOVA revealed main effects of Visual Field (*F* (1, 15) = 7.91, *p* = .013) and Background (*F* (3, 45) = 16.89, *p* < .001). Likewise, the interaction remained significant (*F* (3, 45) = 6.11, *p* = .001). The presence of this interaction confirms that the top ring had a stronger influence than the bottom one. Complementing the ANOVA, the MLE analysis revealed that textures provided more reliable information for both the top (weight _Texture_ = .52, weight _Linear_ = .48) and bottom (weight _Texture_ = .57, weight _Linear_ = .43) rings. Correlations between the observed and predicted estimates were significant for the top (*r* (14) = .61, *p* = .012) and bottom (*r* (14) = .84, *p* < .001) rings (See S2 Fig in Supplementary Materials).

#### Eye-tracking

**Fig 5:**
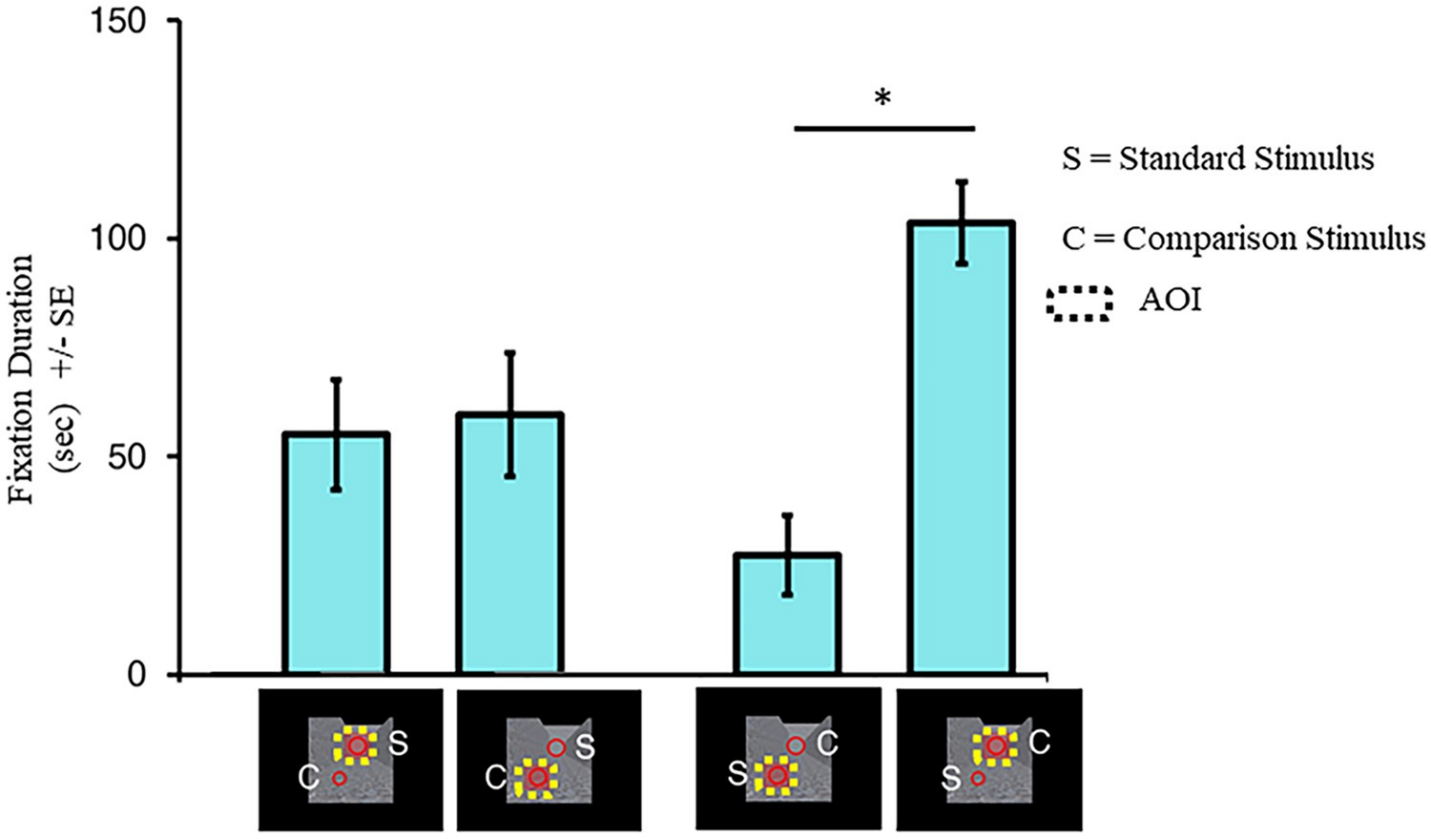
Fixation durations in experiment 1. Fixation durations are the total amount of time participants gazed at an AOI across all trials for a particular condition. The asterisk (*) represents a significant difference at *p* < .05 after a Tukey’s HSD correction was made for multiple comparisons. Error bars represent the standard errors around the mean for within subjects contrasts. These error bars were calculated using procedures described by O’Brien and Cousineau [33].

Fig 5 shows the fixation durations for each AOI for the top and bottom standard ring blocks. An interaction was observed between AOI and Visual Field (*F* (1, 7) = 8.50, *p* = .022). All other interactions did not reach significance (all *p* ≥ .152). There was a main effect of AOI (*F* (1, 7) = 7.69, *p* = .028) but not for Visual Field (*F* (1, 7) = 2.23, *p* = .179) or Background (*F* (3, 21) = 1.18, *p* = .341). Post-hoc Tukey’s HSD pairwise comparison tests showed that participants attended to the top comparison ring more than the bottom standard ring (*p* = .016). There were no differences between fixation durations for the top standard and bottom comparison ring (*p* = .994). Thus, participants fixated on the top comparison ring more than the bottom standard ring when asked to perceptually judge the size of the latter.

#### Additional analyses

Not all participants had eye-tracking. The question then arises whether or not the participants with eye-tracking are representative of those who did not. For the purposes of verification, we performed a series of independent samples *t*-tests on the PSE values for each condition (Table 1). Bonferroni corrections were applied to these tests to account for eight comparisons. These additional tests revealed that there were no differences between the participants who had eye-tracking versus those who did not (all *p* ≥ .272).

We performed an additional ANOVA to determine if the duration of trials differed between conditions. As a reminder, each trial ended when participants made a response. The validity of some of the analyses above depends on an evenly matched duration of trials across the different conditions. ANOVA did not reveal main effects of Visual Field (*F* (1, 7) = .903, *p* = .374) or Background (*F* (3, 21) = .333, *p* = .802). Likewise, the interaction did not reach significance between the two factors (*F* (3, 21) = 2.501, *p* = .087). Thus, trial durations did not differ between conditions. The average trial duration was 2.465 secs (*SD* = 0.154).

### Experiment 2

#### Points of subjective equality (PSEs)

**Fig 6:**
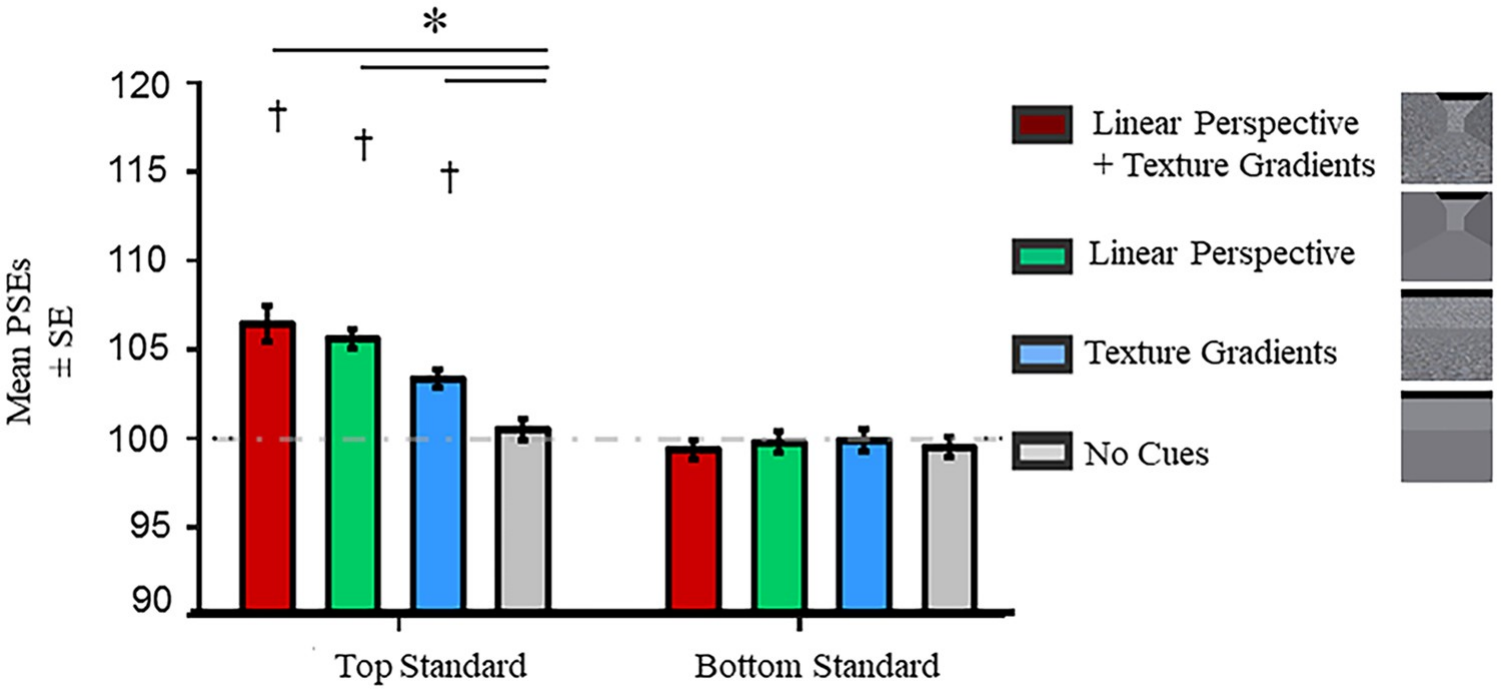
PSEs in experiment 2. Asterisks (*) represent significant differences at *p* < .05 after Tukey’s HSD corrections were made for multiple comparisons. Daggers (†) represent significant differences from the physical size (100 pixels) of the standard ring at *p* < .05 after Bonferroni corrections were made for multiple comparisons. The horizontal dashed line denotes the physical size of the standard ring. PSEs were computed from psychometric functions that best fit the data. Error bars represent standard errors around the mean for within-subjects contrasts. These error bars were calculated using procedures described by O’Brien and Cousineau [33].

Fig 6 shows the mean PSEs for each background for the top and bottom standard ring blocks. Table 2 provides descriptive statistics. One sample *t*-tests revealed that the top standard ring was perceived larger than its physical size (100 pixels) across all backgrounds with pictorial depth cues (all *p* ≤ .008) while the bottom standard ring was not perceived differently than its physical size on any background (all *p* > .999). The direction of the significant shifts in PSEs for the top ring was consistent with what is expected for the Ponzo illusion. Namely, participants perceived the ring to be larger than 100 pixels.

**Table 2:** Descriptive statistics for PSEs in experiment 2. A series of independent samples *t*-tests, which were corrected for multiple comparisons using the Bonferroni method (*p*_corr_), on the PSE values for each condition was performed between the groups of participants with (With Eye-Tacker) and without (No Eye-Tracker) eye-tracking.

	Group	All Participants (N = 16)		No Eye-Tracker (n = 8)		With Eye-Tracker (n = 8)				
		*M*	*SD*	*M*	*SD*	*M*	*SD*	*t*	*p*_uncorr_	*p*_corr_
**Top Ring**	Linear+Texture	106.6	4.88	104.9	3.83	108.4	5.41	-1.49	.158	> .999
	Texture	103.4	3.52	103	3.46	103.9	3.75	-0.53	.603	> .999
	Linear	105.7	2.53	105.9	1.9	105.5	3.16	0.30	.767	> .999
	No Cues	100.5	3.62	99.9	3.61	101.2	3.76	-0.69	.503	> .999
**Bottom Ring**	Linear+Texture	99.4	2.62	99.6	2.87	99.2	2.53	0.28	.783	> .999
	Texture	99.9	3.24	101.2	3.19	98.7	2.93	1.66	.119	.952
	Linear	99.8	1.85	99.4	1.71	100.2	2.01	-0.92	.374	> .999
	No Cues	99.5	2.99	99.5	3.3	99.6	2.87	-0.04	.972	> .999

As in experiment 1, an interaction was observed between Visual Field and Background (*F* (2, 27) = 13.93, *p* < .001, Greenhouse-Geisser corrected). Main effects of Visual Field (*F* (1, 15) = 29.25, *p* < .001) and Background (*F* (3, 45) = 14.02, *p* < .001) were also significant. To further examine the interaction, we conducted post-hoc Tukey’s HSD pairwise comparison tests. These tests showed that the size of the top ring was consistently perceived larger on backgrounds with depth cues than the one without any cues (all *p* ≤ .007) while the perceived size of the bottom ring did not change with the presence of pictorial depth cues compared to when there were none (all *p* ≥ .999). There was no difference in the perceived size of the bottom rings when placed on the background with only linear perspective cues versus the one with only texture gradients (*p* > .999). Difference in the perceived size of the top rings when placed on the background with only linear perspective cues versus the one with only texture gradients trended towards significance (*p* = .062). Taken together, linear perspective cues and texture gradients affected the perceived size of the top ring while it did not change the perceived size of the bottom ring. S2 Table in the supplementary materials provides results for all pairwise comparisons examined.

We repeated the above ANOVA on the absolute shifts in PSEs (**|**100 –PSE**|**). The ANOVA revealed main effects of Visual Field (*F* (1, 15) = 16.83, *p* < .001) and Background (*F* (3, 45) = 4.83, *p* = .005). Likewise, the interaction remained significant (*F* (3, 45) = 7.60, *p* < .001)–confirming that the top ring had a stronger influence than the bottom one.

The MLE analysis revealed that linear perspective cues provided more reliable information for both the top (weight _Linear_ = .66, weight _Texture_ = 34) and bottom (weight _Linear_ = .75, weight _Texture_ = .25) rings. Correlations between the observed and predicted estimates were significant for the top (*r* (14) = .62, *p* = .011) and bottom (*r* (14) = .78, *p* < .001) rings (See S2 Fig in Supplementary Materials).

#### Eye-tracking

**Fig 7:**
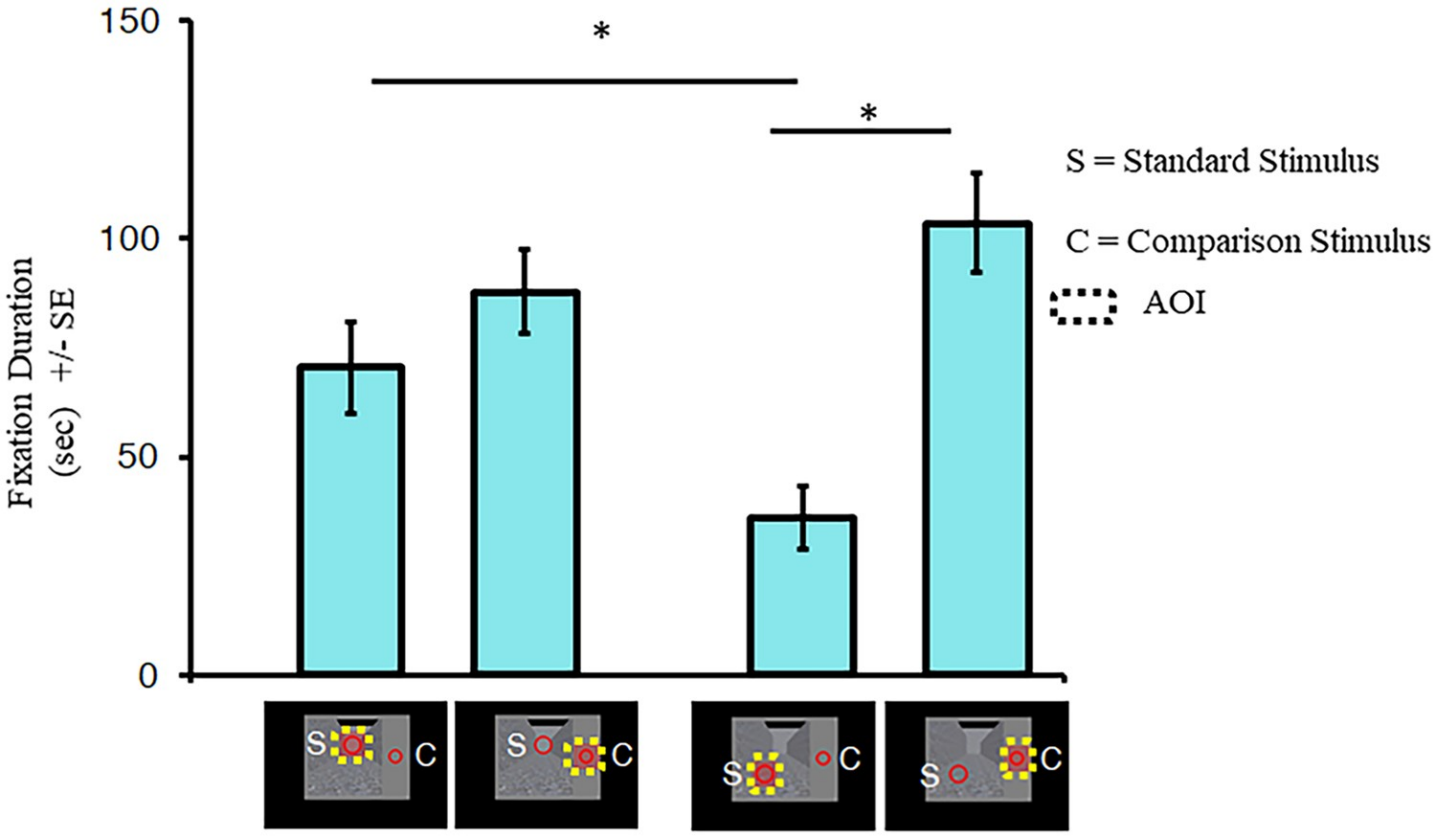
Fixation durations in experiment 2. Fixation durations are the total amount of time participants gazed at an AOI across all trials for a particular condition. The asterisks (*) represent significant differences at *p* < .05 after Tukey’s HSD corrections were made for multiple comparisons. Error bars represent standard errors around the mean for within subjects contrasts. These error bars were calculated using procedures described by O’Brien and Cousineau [33].

Fig 7 shows fixation durations for each AOI for the top and bottom standard ring blocks. The interaction between AOI and Visual Field (*F* (1, 7) = 20.06, *p* = .003) revealed that the effects of AOI changed as a function of where the standard ring was placed in the visual field. Tukey’s HSD pairwise comparison tests revealed that participants spent more time fixating on the top ring compared to the bottom one when they were the standard stimulus (*p* = .016) and that participants spent more time fixating on the comparison stimulus to the side than the bottom ring when the latter was designated as the standard (*p* = .003). In contrast, there were no differences in fixation durations between the standard and comparison AOIs when the top ring was the standard (*p* = 0.214). The interaction between AOI and Background (*F* (3, 21) = 3.67, *p* = .029) was also significant (See S3 Fig in Supplementary Materials). However, Tukey’s HSD pairwise comparison tests failed to confirm this interact but instead revealed that participants spent more time fixating on the comparison compared to the standard ring (all *p* ≤ .002) without any changes across backgrounds for the standard (all *p* ≥ .383) or comparison (all *p* ≥ .269) rings. All other interactions did not reach significance (all *p* ≥ .254). There was a main effect of AOI (*F* (1, 7) = 9.45, *p* = .018) but there were no main effects of Visual Field (*F* (1, 7) = 1.44, *p* = .270) or Background (*F* (3, 21) = .23, *p* = .874).

#### Additional analyses

As in experiment 1, we used independent samples *t*-tests on the PSE values obtained in each condition to verify that participants who had eye-tracking were representative of those who did not (Table 2). Bonferroni corrections were applied to these tests to account for eight comparisons. The tests revealed that there were no differences between the two groups (all *p* ≥ .952).

We performed an additional ANOVA to determine if the duration of trials differed between conditions. ANOVA did not reveal main effects of Visual Field (*F* (1, 7) = 0.701, *p* = .430) and Background (*F* (2,9) = 0.728, *p* = .451, Greenhouse-Geisser corrected). Likewise, the interaction did not reach significance between the two factors (*F* (3, 21) = 0.199, *p* = .896). Thus, trial durations did not differ between conditions. The average trial duration was 2.863 sec (*SD* = 0.434).

## Discussion

The present study investigated the effects of linear perspective cues and texture gradients in the perceptual rescaling of stimuli over a Ponzo-like illusory display of a hallway. We reasoned that the relative contributions of top-down and bottom-up mechanisms in driving the illusion could be inferred from the relative contributions of linear perspective cues and texture gradients. Namely, if the two cues perceptually rescale the stimuli separately and produce an additive effect when they are combined together, then we can infer that top-down mechanisms play an important role. On the other hand, if only a subset of cues perceptually rescales the stimuli, or there is no additive effects, then we can infer that bottom-up mechanisms play an important role in driving the illusion. In experiment 1, we presented both the standard and comparison stimuli over the same background image (Fig 3A and 3B). The presentation of two stimuli over a background is common. However, the perceived size differences in this context can be explained partly by relative size contrast effects [34]. To remove these effects, we performed experiment 2 by presenting the comparison stimulus outside of the background so that only one ring was presented over the background (Fig 3C and 3D). Indeed, the effects of pictorial depth cues on perceptual rescaling were stronger in experiment 1 compared to experiment 2. In addition, experiment 2 revealed that the perceived size of the top but not the bottom ring changed depending on the availability of linear perspective cues and texture gradients.

The present study yielded the following novel findings. First, both linear perspective cues and texture gradients perceptually rescaled the stimuli separately. Second, illusion strength increased when the participants judged the size of the stimulus in the upper visual field where they tended to gaze more. Third, linear perspective and texture gradient cues provided information that were more or less equally reliable when both rings were presented in the background while linear perspective cues provided more reliable information when the comparison stimulus was presented outside the background. As we will discuss, the first two findings provide support for top-down mechanisms while the third finding provides evidence for both top-down and bottom-up mechanisms.

In contrast to the present study, Leibowitz et al. [11] and Fineman and Carlson [12] observed differences between the contributions of linear perspective cues and texture gradients in the magnitude of the illusion. These differences may in part be explained by the fact that Leibowitz et al. [11] evaluated the unique contribution of texture gradients with a background that simulated linear perspective cues as well. By contrast, Fineman and Carlson [12] tested the contribution of texture gradients with a background where linear perspective cues were not presented on realistic textures but rather on ecologically questionable texture patterns. We believe that presenting texture gradients with linear perspective depth cues may have increased the contribution of texture gradients in Leibowitz et al.’s study [11] while using texture gradients that do not look like textures as we see in real life may weaken depth information and affect the contribution of texture gradients to perceptual rescaling mechanisms in Fineman and Carlson’s study [12]. In the present study, we showed that when the contribution of linear perspective cues and texture gradients were tested in a more controlled manner by systematically adding and subtracting them in a background image, texture gradients produced as strong of an illusion as linear perspective cues. In the present investigation, the contribution of linear perspective cues and texture gradients is more likely dependent on the strength of each of the cues than some kind of nuisance variable, which were present in these earlier studies.

We also found that illusion strength was stronger when the participants judged the size of the stimulus in the upper visual field. Namely, the top stimulus in the upper visual field was consistently overestimated on each background with pictorial depth cues as compared to the plain background. However, the size of the bottom stimulus was underestimated only if both the standard and comparison stimuli were presented over the background with linear perspective cues. Note that participants were looking at a two-dimensional surface in this study. Stereopsis, vergence, and accommodation signalled to the participant that the top and bottom rings were the same distance. Therefore, these distance cues are unable to explain why the perceptual rescaling of size is stronger for the top stimulus compared to the bottom one. Instead, a higher degree of attention could have been drawn to the upper visual field because this is where pictorial distance cues are more informative than binocular and oculomotor distance cues in the real world [4].

This idea is supported by the eye-tracking data. To quantify where participants gazed more, we calculated the total fixation durations in each AOI for the standard and comparison stimuli. We could not monitor all participants’ eye positioning due to the availability of the eye-tracker, but we have two reasons to believe that the results of the participants who had eye tracking were representative of all participants. First, we gave the same instructions and task to all participants. The only difference for participants who had eye tracking was the presence of an eye tracker in front of them and a calibration procedure at the beginning of each block. It is unlikely that these differences would affect the results. Second, we compared the magnitude of size illusion for these two groups of participants with independent samples *t*-tests and we could not find any significant differences between them. Thus, the eye tracking data should be representative of all participants.

The results of eye-tracking revealed that the participants tended to gaze more in the upper than the lower visual field. This is particularly evident in experiment 2. In this experiment, participants spent twice as much time fixating on the top comparison stimulus compared to the bottom standard stimulus when they had to judge their size (Fig 7). This is consistent with other studies showing an increase in the strength of the Ponzo illusion [22–24], and other illusions, such as the vertical-horizontal [21] and the Muller-Lyer [23] illusions, with greater attentional focus.

The finding that participants tended to gaze more in the upper compared to the lower visual field is consistent with our predictions. Previously, Miller [35] argued that the linear perspective cues in corridors frequently draw our attention towards the upper visual field in everyday life and suggested that this experience contributes to the strength of the Ponzo illusion. Miller [35] tested this prediction by presenting the illusion in different orientations. The author found that the magnitude of Ponzo illusion was stronger when the contextual lines converged in the upper visual field, as is the case in its typical configuration, compared to when these lines converged in the lower visual field when the entire display is rotated by 180 degrees. In fact, there was a strong illusion for the former and no illusion for the latter. Our observations are in line with Miller’s findings [35] showing that previous experience with how pictorial depth cues inform the brain how far objects are affects where we attend mostly and influence our size judgments.

Our results also demonstrate that directing attention toward the upper visual field does not result in perceiving the top stimulus as larger on the background without pictorial depth cues. Therefore, attending to the upper visual field does not affect size perception by itself. The presence of the depth cues is necessary to observe the additive effects of attentional mechanisms that increase the contribution of available pictorial depth cues to perceptual rescaling. This is an important consideration. Others have shown that stimuli appear larger in the fovea than in the periphery [36, 37]. This could potentially explain the Ponzo illusion if one considers that the linear perspective cues are directing people’s gaze to the top stimulus, which appears larger than the bottom one. However, the participants in this study tended to also fixate more in the upper visual field without the linear perspective cues. This did not result in a perceptual rescaling of size in the no-cue condition. Hence, low-level explanations, such as where the eyes are attending to, cannot explain the illusory effects we report in this study.

However, our MLE results are counterintuitive. Namely, we found that texture gradients were as reliable as linear perspective cues in experiment 1 in which the standard and comparison stimuli were placed over the different sections of the same background. This finding supports the contribution of top-down mechanisms [38–40], showing that both linear perspective cues and texture gradients contribute more or less equally to perceptual rescaling. In this instance, an equal consideration of both cues is suggestive of higher-order processing considering different sources of information. Contrarily, we found that linear perspective cues were much more reliable than texture gradients in experiment 2 in which the comparison stimulus was placed outside of the background, which is suggestive of bottom-up mechanisms [41, 42]. In this instance, a greater consideration of one cue over the other is suggestive of one bottom-up channel being favoured over another. Bottom-up accounts for the Ponzo illusion propose that the nearby contextual elements surrounding the top and bottom stimuli cause differences in how each one is perceived. In addition, the differences between the reliabilities of linear perspective cues and texture gradients is interesting in light of Rennig et al.’s study [13] demonstrating how Kanizsa triangles over a Ponzo-like display appear to have different sizes when linear perspective but not texture gradients are provided. These differential effects suggest that the manner with which contextual elements surround each stimulus is important for their perceptual rescaling–which is more in line with bottom-up than top-down explanations of the Ponzo illusion.

In conclusion, the present study shows that both linear perspective cues and texture gradients contribute to perceptual rescaling mechanisms in the context of the Ponzo illusion. Moreover, we found that the effects of the two pictorial depth cues to perceptual rescaling mechanisms were stronger in the visual field where participants directed their attention. Our findings suggest that both top-down and bottom-up mechanisms play an important role in the Ponzo illusion. However, the former seems to fulfil a more prominent role when both stimuli are present in the illusory background.

## Supporting information

S1 Fig The mean PSE curves with 95% CIs across backgrounds for the top and the bottom standard ring blocks in experiments 1 and 2. Proportions of responses where the participants perceived the comparison ring as larger than the standard at each increment were plotted against the physical size range of the comparison ring to fit a psychometric function.
(TIF)

S2 Fig Correlations between Observed and Predicted PSEs. Observed PSEs represent each participants’ PSE measurements for linear perspective + texture background. Predicted PSEs represent each participants’ weighted linear summation of PSE measurements for the linear perspective and texture backgrounds.
(TIF)

S3 Fig Fixation durations across backgrounds in experiment 2. Fixation durations are the total amount of time participants gazed at an AOI across all trials for each background. The asterisks (*) represent significant differences at *p* < .05 after Tukey’s HSD corrections were made for multiple comparisons. Error bars represent standard errors around the mean for within subjects contrasts. These error bars were calculated using procedures described by O’Brien and Cousineau [33].
(TIF)

S1 Table Results for all post-hoc Tukey’s HSD pairwise comparison tests on PSEs in experiment 1. (DOCX)

S2 Table Results for all post-hoc Tukey’s HSD pairwise comparison tests on PSEs in experiment 2. (DOCX)
