# Supplementary figures and images for: The contribution of linear perspective cues and texture gradients in the perceptual rescaling of stimuli inside a Ponzo illusion corridor
Source: PLoS One. 2019 Oct 10;14(10):e0223583. doi: 10.1371/journal.pone.0223583 (PMC6786755; doi:10.1371/journal.pone.0223583)

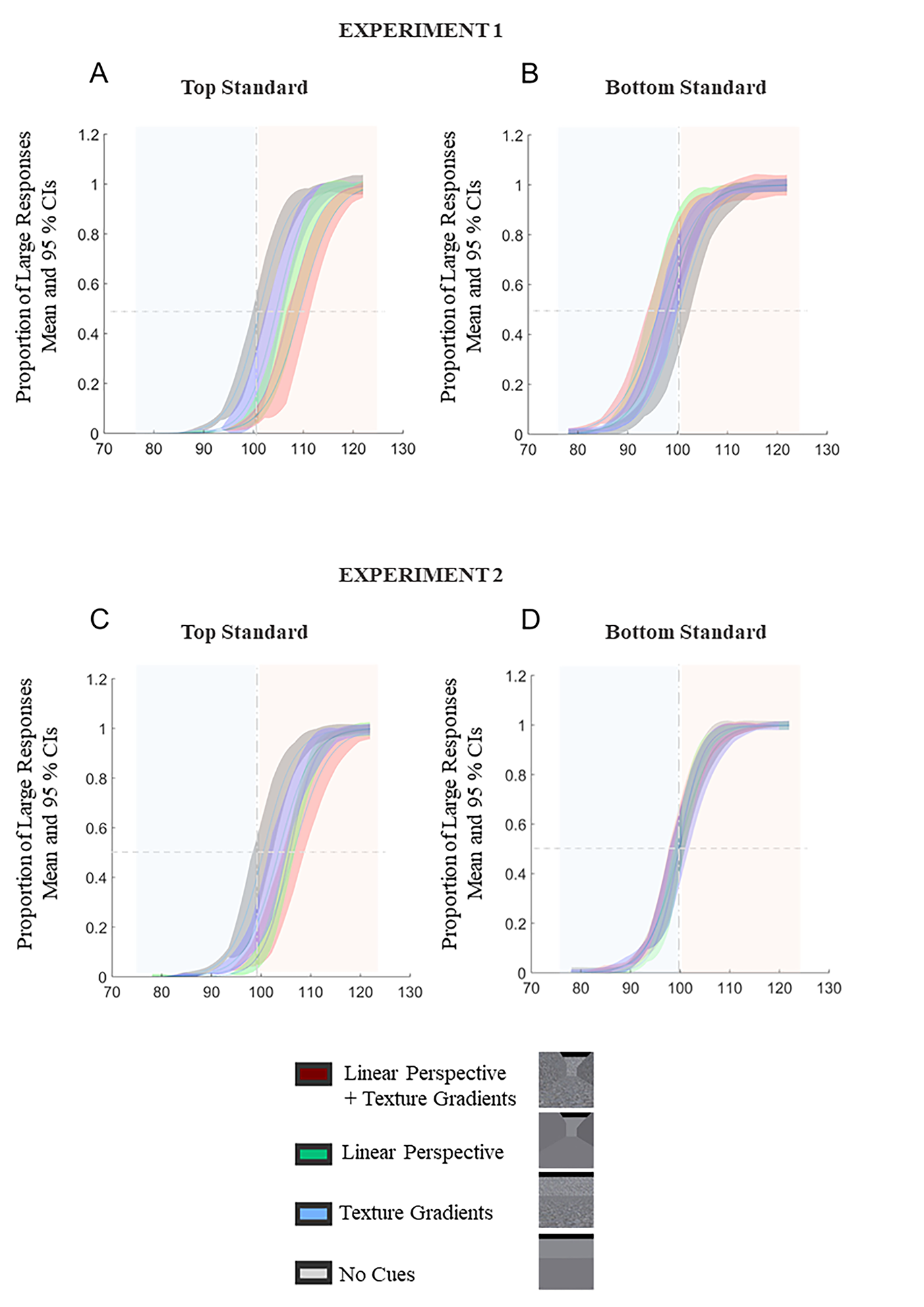

Supplement: S1 Fig — Proportions of responses where the participants perceived the comparison ring as larger than the standard at each increment were plotted against the physical size range of the comparison ring to fit a psychometric function. (TIF) [file pone.0223583.s001.tif]

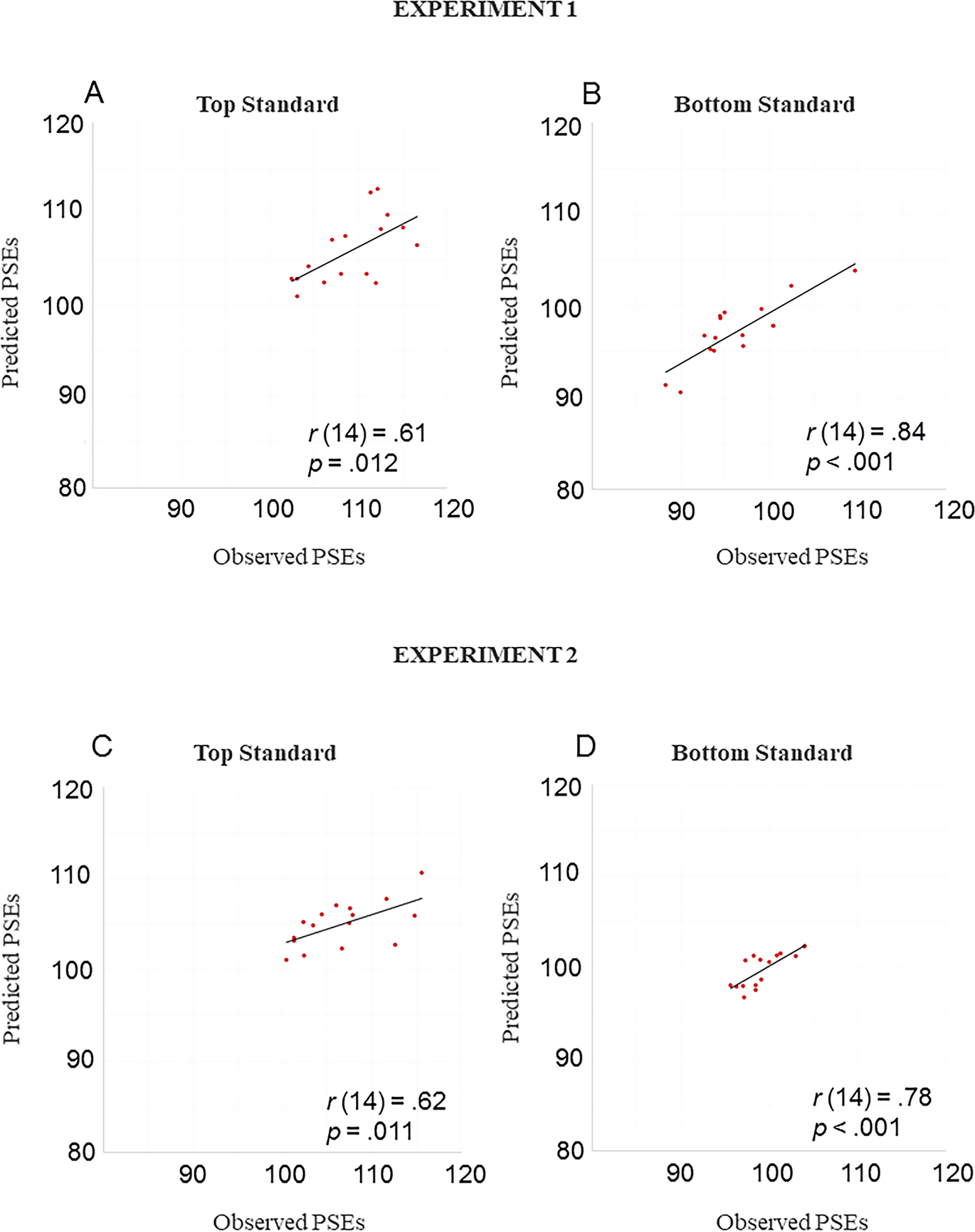

Supplement: S2 Fig — Observed PSEs represent each participants’ PSE measurements for linear perspective + texture background. Predicted PSEs represent each participants’ weighted linear summation of PSE measurements for the linear perspective and texture backgrounds. (TIF) [file pone.0223583.s002.tif]

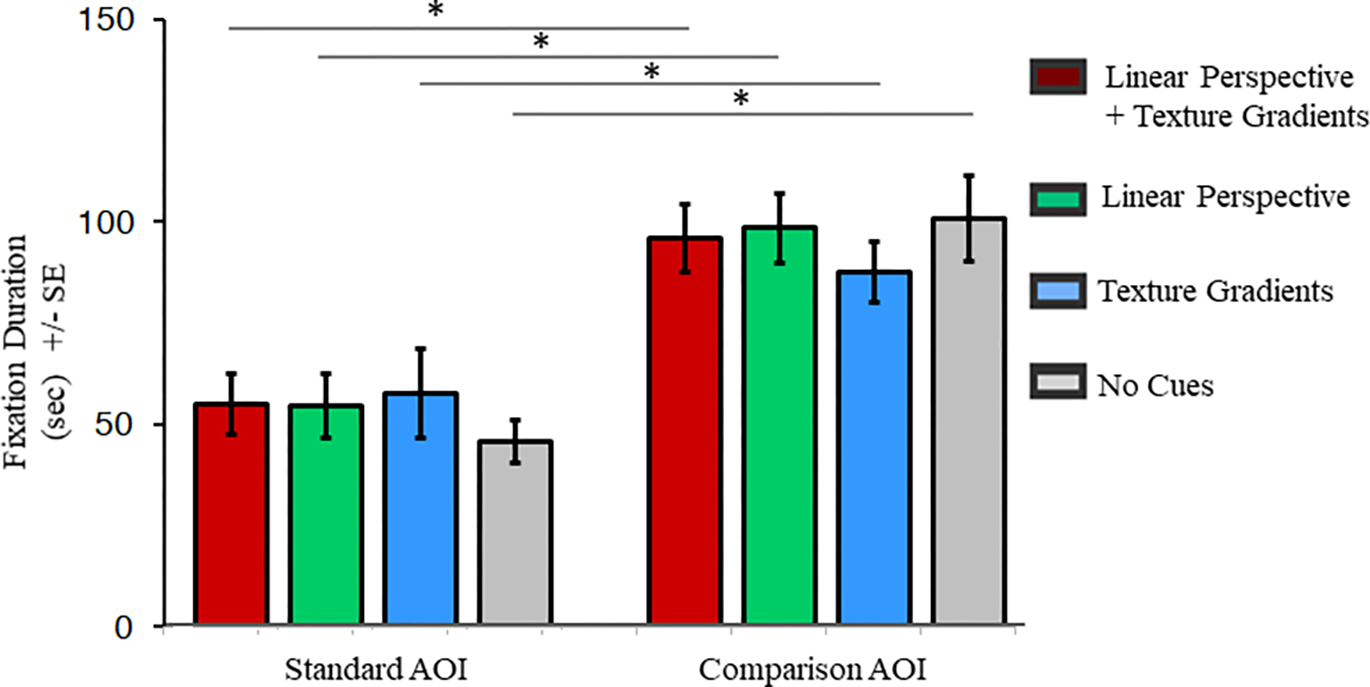

Supplement: S3 Fig — Fixation durations are the total amount of time participants gazed at an AOI across all trials for each background. The asterisks (*) represent significant differences at p < .05 after Tukey’s HSD corrections were made for multiple comparisons. Error bars represent standard errors around the mean for within subjects contrasts. These error bars were calculated using procedures described by O’Brien and Cousineau [33]. (TIF) [file pone.0223583.s003.tif]
