# Supplementary material for: The contribution of linear perspective cues and texture gradients in the perceptual rescaling of stimuli inside a Ponzo illusion corridor
Source: PLoS One. 2019 Oct 10;14(10):e0223583. doi: 10.1371/journal.pone.0223583 (PMC6786755; doi:10.1371/journal.pone.0223583)
Supplement: S1 Table — (DOCX) [file pone.0223583.s004.docx]

|  |  |  | **Top Ring** | | | **Bottom Ring** | | | |
| --- | --- | --- | --- | --- | --- | --- | --- | --- | --- |
|  |  |  | Texture | Linear | No Cues | Linear +  Texture | Texture | Linear | No Cues |
| **Top Ring** | Linear + Texture | *t* (15) | **4.11** | 1.97 | **6.35** | 7.02 | 6.74 | 7.67 | 6.07 |
|  |  | *p_corr_* | **.005*** | 0.559 | **.001*** | .001* | .001* | .001* | .001* |
|  | Texture Gradients |  | *t* (15) | -2.43 | **3.79** | 6.04 | 4.91 | 6.94 | 3.27 |
|  |  |  | *p_corr_* | 0.394 | **.028*** | .001* | .001* | .001* | .005* |
|  | Linear Perspective |  |  | *t* (15) | **6.18** | 6.99 | 6.45 | 7.71 | 6.05 |
|  |  |  |  | *p_corr_* | **.001*** | .001* | .001* | .001* | .001* |
|  | No Cues |  |  |  | *t* (15) | 4.07 | 3.4 | 5.07 | 0.81 |
|  |  |  |  |  | *p_corr_* | .002* | 0.053 | .001* | 0.998 |
| **Bottom Ring** | Linear + Texture |  |  |  |  | *t* (15) | -1.61 | 0.22 | **-3.76** |
|  |  |  |  |  |  | *p_corr_* | 0.81 | > .999 | **.006*** |
|  | Texture Gradients |  |  |  |  |  | *t* (15) | 2.29 | -3.49 |
|  |  |  |  |  |  |  | *p_corr_* | 0.743 | 0.221 |
|  | Linear Perspective |  |  |  |  |  |  | *t* (15) | **-4.22** |
|  |  |  |  |  |  |  |  | *p_corr_* | **.005*** |

Asterisks (*) represent significant differences at *p* < .05 after Tukey’s HSD corrections were made for multiple comparisons.
