# Supplementary material for: The contribution of linear perspective cues and texture gradients in the perceptual rescaling of stimuli inside a Ponzo illusion corridor
Source: PLoS One. 2019 Oct 10;14(10):e0223583. doi: 10.1371/journal.pone.0223583 (PMC6786755; doi:10.1371/journal.pone.0223583)
Supplement: S2 Table — (DOCX) [file pone.0223583.s005.docx]

|  |  |  | **Top Ring** | | | **Bottom Ring** | | | |
| --- | --- | --- | --- | --- | --- | --- | --- | --- | --- |
|  |  |  | Texture | Linear | No Cues | Linear + Texture | Texture | Linear | No Cues |
| **Top Ring** | Linear + Texture | *t* (15) | **3.48** | 0.81 | **6.2** | 5.21 | 4.46 | 5.14 | 5.71 |
|  |  | *p_corr_* | **.00*** | 0.93 | **.00*** | .00* | .00* | .00* | .00* |
|  | Texture Gradients |  | *t* (15) | -2.96 | **5.48** | 5.06 | 4.13 | 3.82 | 4.66 |
|  |  |  | *p_corr_* | 0.062 | **.007*** | .001* | .001* | .001* | .001* |
|  | Linear Perspective |  |  | *t* (15) | **8.21** | 8 | 6.87 | 7.33 | 6.57 |
|  |  |  |  | *p_corr_* | **.001*** | .001* | .001* | .001* | .001* |
|  | No Cues |  |  |  | *t* (15) | 1.3 | 0.65 | 0.7 | 1.01 |
|  |  |  |  |  | *p_corr_* | 0.762 | 0.991 | 0.976 | 0.872 |
| **Bottom Ring** | Linear +  Texture |  |  |  |  | *t* (15) | -1.13 | -0.83 | -0.28 |
|  |  |  |  |  |  | *p_corr_* | 0.995 | 0.999 | > .999 |
|  | Texture Gradients |  |  |  |  |  | *t* (15) | 0.15 | 0.6 |
|  |  |  |  |  |  |  | *p_corr_* | >.999 | 0.999 |
|  | Linear Perspective |  |  |  |  |  |  | *t* (15) | 0.51 |
|  |  |  |  |  |  |  |  | *p_corr_* | > .999 |

Asterisks (*) represent significant differences at *p* < .05 after Tukey’s HSD corrections were made for multiple comparisons.
